# Cardiac Output Values and Correlation of Renal Injury with Neutrophil Gelatinase-Associated Lipocalin Levels in Off-Pump Coronary Artery Bypass Patients

**DOI:** 10.21470/1678-9741-2019-0467

**Published:** 2020

**Authors:** Şahin Şahinalp, Zeki Temiztürk, Kadir Çeviker, Kanat Özışık, Uğursay Kızıltepe

**Affiliations:** 1 Department of Cardiovascular Surgery, Faculty of Medicine, Van Yuzuncu Yil University, Van, Turkey.; 2 Department of Cardiovascular Surgery, Elazig Education and Research Hospital, University of Health Sciences, Elazig, Turkey.; 3 Department of Cardiovascular Surgery, Western Anatolia Central Hospital, Izmir, Turkey.; 4 Department of Cardiovascular Surgery, Ankara City Hospital, Ankara, Turkey.; 5 Department of Cardiovascular Surgery, Diskapi Yildirim Beyazit Education and Research Hospital, University of Health Sciences, Ankara, Turkey.

**Keywords:** Coronary Artery Bypass, Off-Pump, Anastomosis, Surgical, Coronary Vessels, Arterial Pressure, Cardiac Output, Biomarkers, Lipocalin-2, Longitudinal Studies

## Abstract

**Objective:**

To investigate the correlation between cardiac output values and renal neutrophil gelatinase-associated lipocalin (NGAL) levels as a biomarker of renal ischemia.

**Methods:**

Forty patients, who underwent off-pump coronary artery bypass (OPCAB) surgery and in whom the positioning of the heart was fixed with simple suspension sutures without a mechanical stabilizer, were included in the study. Continuous cardiac output (CO) measurements were recorded using the arterial pressure waveform analysis method (FloTrac sensor system) in the perioperative period. CO was recorded every minute during non-anatomical cardiac positioning for left anterior descending artery (LAD), diagonal artery (D), circumflex artery (Cx), and right coronary artery (RCA) bypasses. Serum NGAL samples were analyzed in the preoperative, perioperative, and postoperative periods.

**Results:**

The CO values measured at various non-anatomical cardiac positions during distal anastomosis for LAD, D, Cx, and RCA were significantly lower than pre- and postoperative values measured with the heart in normal anatomical position (3.45±0.78, 2.9±0.71, 3.11±0.56, 3.19±0.81, 5.03±1.4, and 4.85±0.78, respectively, *P*=0.008). There was no significant difference between CO values measured at various non-anatomical cardiac positions during distal anastomosis. Although there was no significant correlation between NGAL levels and age, duration of surgery, preoperative CO, D-CO, RCA-CO, and postoperative CO measurements, there was a significant correlation between NGAL levels and LAD-CO (*P*=0.044) and Cx-CO (*P*=0.018) at the postoperative 12^th^ hour.

**Conclusion:**

Full revascularization may be achieved by employing the OPCAB technique while using simple suspension sutures without a mechanical stabilizer and by providing safe CO levels and low risk of renal ischemia.

**Table:** 

Abbreviations, acronyms & symbols				
ANOVA	= Analysis of variance		EuroSCORE	= European System for Cardiac Operative Risk Evaluation
BMI	= Body mass index		LAD	= Left anterior descending artery
CABG	= Coronary artery bypass grafting		LIMA	= Left internal mammary artery
CO	= Cardiac output		NGAL	= Neutrophil gelatinase-associated lipocalin
COPD	= Chronic obstructive pulmonary disease		OPCAB	= Off-pump coronary artery bypass
CPB	= Cardiopulmonary bypass		RCA	= Right coronary artery
Cx	= Circumflex artery		SD	= Standard deviation
D	= Diagonal artery			
EF	= Ejection fraction			

## INTRODUCTION

Although discussions concerning the merits and demerits of on/off-pump coronary artery bypass grafting (CABG) are ongoing, off-pump coronary artery bypass (OPCAB) surgery is frequently performed in many surgical centers. The main limitations of OPCAB technique are hemodynamic alterations that may be caused by factors such as the different stabilization methods used, interruption of coronary blood flow, and positioning of the heart in non-anatomical positions. In cases where OPCAB surgery was performed, adverse hemodynamic changes, both in the use of stabilizer devices and in deep pericardial sling methods, have been observed in a number of studies^[1-3]^. Cardiac output (CO) is one of the most important parameters for the evaluation of these undesired hemodynamic changes. The arterial pressure waveform analysis method (using FloTrac sensor system) was shown to continuously measure CO within reasonable margins of error^[4,5]^. By closely monitoring the hemodynamic parameters and limiting their alterations, complications involving vital organs may be reduced, thus directly affecting the success of OPCAB. An important complication of these hemodynamic alterations is acute renal failure, which is one of the major causes of mortality after CABG surgery (including OPCAB), and may occur at varying frequencies^[6-8]^. Neutrophil gelatinase-associated lipocalin (NGAL) is a biochemical marker that spikes much earlier in the serum and urine samples in the event of renal ischemia or exposure to nephrotoxic agents compared to serum urea and creatinine levels^[9]^. NGAL is therefore a biomarker that can be used for the early prediction of acute renal failure^[9]^. Several studies have reported a relationship between elevated NGAL levels and the development of acute renal failure following cardiac surgery^[6,9]^.

The aims of this study were to determine changes in CO level in patients undergoing OPCAB surgery using simple suspension sutures without a mechanical stabilizer and to investigate the relationship between CO and serum NGAL levels. The reliability of OPCAB surgery employing the epicardial sling technique in the context of risk of development of associated renal ischemia can thus be better understood.

## METHODS

### Patient Selection

Forty patients (female:male ratio = 1:3, mean age: 60.2 ± 10.2 years) who underwent OPCAB surgery between February 2011 and May 2011 were included in the study and evaluated prospectively. Heart valve patients requiring further intervention in addition to CABG, patients undergoing additional procedures requiring cardiopulmonary bypass (CPB), as well as patients diagnosed with acute or chronic renal failure were excluded from the study. Patients were included in the study regardless of the presence of angiographic lesions or the number of coronary arteries requiring bypass. No additional criteria for performing OPCAB were sought. All participants were provided with detailed information regarding the study and informed consent was obtained for surgery. This study was conducted with the approval of the local institutional ethics committee and adhered to the principles of the Declaration of Helsinki. The patients underwent preoperative routine anamnesis and physical examination, and their laboratory results were evaluated prior to surgery.

### Anesthesia and Surgical Procedure

All participants were prepared for routine CABG procedure, monitored by intraoperative electrocardiography, and assessed for invasive arterial pressure, central venous pressure, pulse oximetry, and body temperature.

Continuous CO monitoring was provided to all patients by means of a noninvasive method using the FloTrac sensor system (FloTrac Vigileo System, Edwards Lifesciences, Inc., Nyon, Switzerland). This system was connected to the line through which invasive arterial pressure was monitored, and continuous measurements were taken.

All patients underwent a combined approach in which both inhalation and intravenous narcotic anesthesia techniques were employed. Anesthesia was induced by means of fentanyl citrate (5-10 µg/kg), thiopental (3-5 mg/kg), or propofol infusion (3-4 mg/kg/hour). Fentanyl, propofol, and low concentrations of sevoflurane were used to maintain anesthesia.

Following median sternotomy, the left internal mammary artery (LIMA) was prepared simultaneously with the radial artery and saphenous vein grafts in the appropriate patients. Anastomosis was carried out after the preparation of the grafts and heparinization (activated clotting time > 200 seconds). During the anastomosis period, an intravenous beta-blocker (esmolol or metoprolol) was administered as needed to achieve a heart rate of 50-60 beats/minute. In order to maintain main a blood pressure of 50-70 mmHg during distal anastomosis, the operating table was positioned at Trendelenburg position and/or dose titration of volatile anesthetics was carried out. The priority was generally given to left anterior descending artery (LAD) anastomosis in patients with proximal and critical lesions or left main coronary artery lesions during distal anastomosis. Revascularization of the other coronary artery was subsequently achieved. The goal was thus to ensure sufficient coronary perfusion during manipulations to reach the posterior and lateral sides of the heart. No intracoronary shunt was used in any patient. Details regarding the technique of exposure and stabilization methods have been described by Kurtoglu et al.^[3]^ in the literature. Deep pericardial and epicardial suspension sutures were used to reach the anastomosis site and provide stabilization. No stabilizer was used in any patient because of possible adverse effects on systemic hemodynamics. Opening of the right pleura was not routinely used to position the posterolateral region of the heart. To improve visualization of the anastomosis site, warm physiological serum was used. During distal anastomosis, bleeding from the target coronary artery was controlled using atraumatic bulldog clamps. Proximal anastomoses were performed on the ascending aorta for all patients. The proximal ends of the radial artery grafts were anastomosed on the LIMA in appropriate patients. None of the patients were administered protamine.

### CO and NGAL Sampling

CO measurements were carried out with the aid of the FloTrac sensor system (FloTrac Vigileo System, Edwards Lifesciences, Inc., Nyon, Switzerland) after invasive arterial cannula placement in the radial artery and recorded as preoperative CO at five-minute intervals from the time of the cannula insertion to the start of distal anastomosis. After the appropriate cardiac position was secured for the distal anastomosis for each coronary artery, measurements were taken at one-minute intervals until completion of anastomosis and recorded as LAD-CO, diagonal artery (D)-CO, circumflex artery (Cx)-CO, and right coronary artery (RCA)-CO. Following the normal anatomic positioning of the heart after distal anastomosis was completed, measurements were taken at five-minute intervals until the end of the surgery and were recorded as postoperative CO.

Peripheral venous blood samples were taken prior to induction of anesthesia (T1) and postoperatively at the 1^st^, 12^th^, 24^th^, and 48^th^ hours to determine serum NGAL levels; each interval was coded as T1, T2, T3, T4, or T5, respectively. Serum samples obtained after centrifugation of the blood samples were stored at -80 ºC until use. NGAL levels were measured with a human lipocalin-2/NGAL enzyme-linked immunosorbent assay kit (BioVendor Laboratorni Medicina, Inc., Czech Republic).

Samples to measure serum urea and creatinine levels were collected prior to induction of anesthesia (T1) and postoperatively at the 24^th^ and 48^th^ hours by the peripheral venous route and recorded as T1, T4, and T5, respectively. Plasma creatinine levels were analyzed using the enzymatic CREA-plus assay method and blood urea nitrogen levels were determined using the kinetic ultraviolet assay (BEN Biochemical Enterprise, Milan, Italy) on a ChemWell 2910 analyzer (Awareness Technology, Inc., Palm City, United States of America).

### Statistical Analysis

Statistical analyses were performed using the IBM SPSS Statistics software, version 21.0. The Kolmogorov-Smirnov normality test was used to assess the data for normal distribution prior to analysis, while Levene’s test was used to evaluate homogeneous variance assumption. One-way analysis of variance (ANOVA) was used to compare patient CO values measured in the LAD, D, Cx, and RCA cardiac positions, and in case of significant values, the Bonferroni test was employed for binary comparisons. One-way ANOVA for repeated measurements was used to analyze NGAL, urea, and creatinine values. Regression analysis and correlation analysis were used to investigate the effect of CO values measured in the four cardiac positions and the duration of each position on NGAL measurements. Repeated measures ANOVA was also employed to analyze the effects of urine volume, duration of surgery, CO values, and postoperative NGAL values. The P-values obtained in the test results were evaluated at a significance level of α = 0.05.

## RESULTS

A total of 40 patients undergoing OPCAB surgery participated in the study, including 10 women (25%) and 30 men (75%). Demographic data, nature of the disease, and surgical characteristics of all patients are presented in Table 1.

**Table 1:** Demographic characteristics of the study patients.

	Mean ± SD
Age (years)	60±10
Male gender (%)	75
BMI (kg/m^2^)	26.02±4.42
Diabetes mellitus (%)	35
Smoker (%)	55
COPD (%)	22.5
EuroSCORE	2.26±1.21
EF (%)	50.67±12.96
Urea (mg/dl)	42.98±20.01
Creatinine (mg/dl)	0.97±0.64
Graft count (n)	3±1
Operation time (minutes)	296±68
Urine output during operation (L)	2.08±0.98

BMI=body mass index; COPD=chronic obstructive pulmonary disease; EF=ejection fraction; EuroSCORE=European System for Cardiac Operative Risk Evaluation; SD=standard deviation

**Fig. 1:**
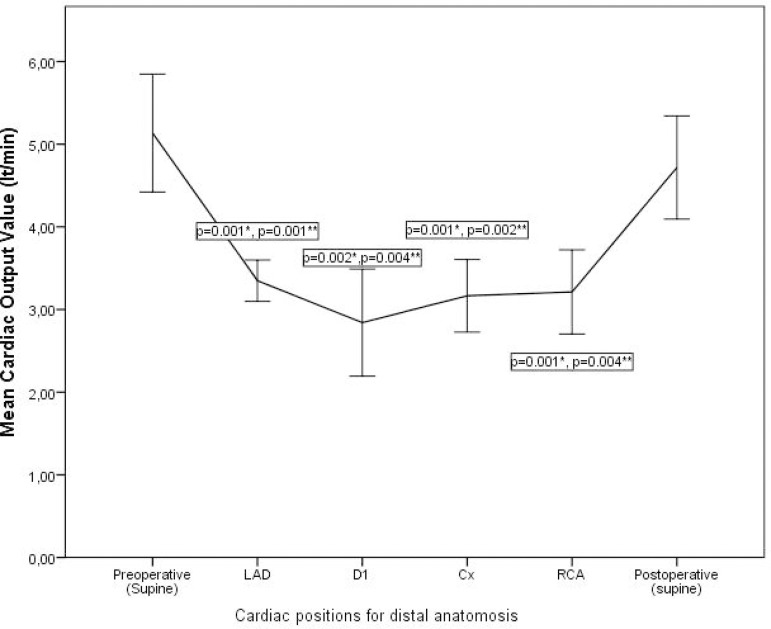
Mean cardiac output (CO) values measured at different cardiac positions. Cx=circumflex artery; D1=diagonal artery; LAD=left anterior descending artery; RCA=right coronary artery. *Repeated measures analysis of variance (ANOVA) test showing statistical significance (P<0.05) in relation to the preoperatively measured CO. **Repeated measures ANOVA test showing statistical significance (P<0.05) in relation to the postoperatively measured CO.

When the CO values measured at different cardiac positions were compared, the preoperative (*) and postoperative (**) CO values were found to be significantly different (*P*<0.05) based on intragroup comparisons with the Bonferroni test (Figure 1). The CO values measured at the different heart positions during distal anastomosis for LAD, D, Cx, and RCA were significantly lower than pre- and postoperative CO values obtained with the heart in the normal anatomical position (3.45±0.78, 2.9±0.71, 3.11±0.56, 3.19±0.81, 5.03±1.4 and 4.85±0.78, respectively). There was no significant difference between CO values measured at LAD, D, Cx, and RCA positions during distal anastomosis (Figure 1).

**Fig. 2:**
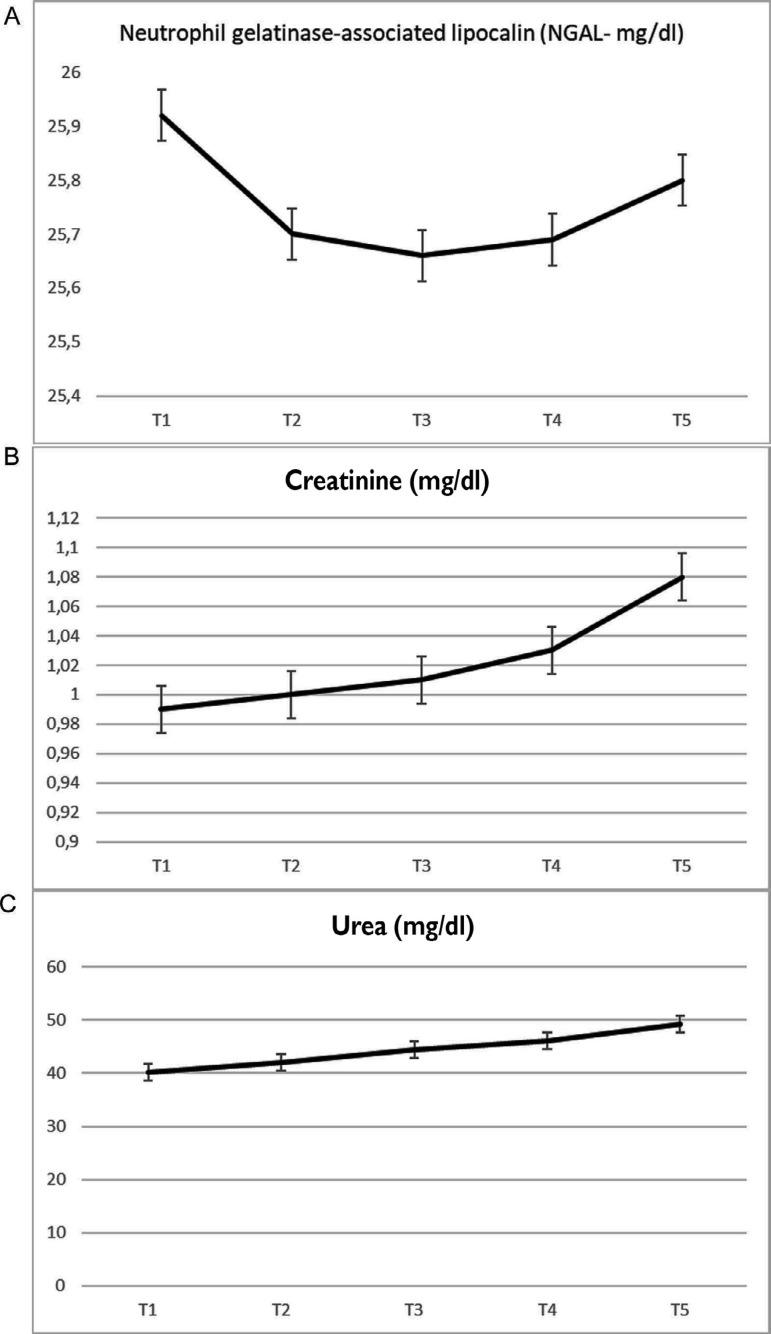
Temporal evaluation of the mean value of serum neutrophil gelatinase-associated lipocalin (NGAL) (A), creatinine (B), and urea (C) in off-pump coronary artery bypass graft surgery. T1=before induction; T2=postoperative 1st hour; T3=postoperative 12th hour; T4=postoperative 24th hour; T5=48th postoperative hour. Statistical analysis was carried out by one-way analysis of variance for repeated measurements to analyze NGAL, urea, and creatinine, and all of the values were found to be statistically non-significant.

Although high levels of NGAL were detected in the postoperative 24^th^ hour, the difference was not statistically significant (Figure 2, *P*=0.74). Univariate analysis of the NGAL levels according to age, duration of surgery, urine volume, and CO in the various cardiac positions during surgery are presented in Table 2. Correlation analyses between the NGAL levels and age, duration of surgery, urine volume, preoperative CO, CO during D anastomosis, and CO during RCA anastomosis, or postoperative CO measurements were not found to be statistically significant (Table 2). On the other hand, a significant association was found between NGAL levels and CO during LAD anastomosis (*P*=0.04) and CO during Cx anastomosis (*P*<0.01) at the postoperative 12^th^ hour (Table 2).

**Table 2:** Univariate analysis of the neutrophil gelatinase-associated lipocalin (NGAL) according to age, duration of surgery, urine volume, and cardiac output in various cardiac positions during surgery.

	NGAL									
	T1		T2		T3		T4		T 5	
	Adjusted R squared	*P*-value	Adjusted R squared	*P*-value	Adjusted R squared	*P*-value	Adjusted R squared	*P*-value	Adjusted R squared	*P*-value
Age (years)	-0.008	.412	-0.019	.597	-0.008	.405	0.002	.304	-0.006	0.387
Operation time	0.069	.056	-0.014	.499	-0.025	.823	0.023	.175	-0.020	0.639
Urine volume	-0.026	.892	-0.017	.554	-0.026	.931	-0.001	.337	0.082	0.042
Preoperative CO	-0.026	.878	-0.009	.431	0.006	.27	0.024	.169	0.022	0.179
CO during LAD anastomosis	0.003	.302	-0.026	.732	-0.022	.631	0.03	.158	0.088	0.044
CO during D anastomosis	-0.005	.354	-0.088	.861	0.019	.291	-0.076	.703	-0.053	0.544
CO during Cx anastomosis	0.036	.182	-0.039	.751	0.09	.08	0.141	.036	0.187	0.018
CO during RCA anastomosis	0.056	.15	0.073	.12	0.043	.18	-0.042	.704	-0.039	0.647
Postoperative CO	-0.008	.412	-0.025	.818	-0.017	.558	-0.009	.425	-0.026	0.926

T1=prior to induction of anesthesia; T2=postoperative 1^st^ hour; T3=postoperative 12^th^ hour; T4=postoperative 24^th^ hour; and T5=postoperative 48^th^ hour. Statistical significance: *P*<0.05 CO=cardiac output; Cx=circumflex artery; D=diagonal artery; LAD=left anterior descending artery; RCA=right coronary artery

## DISCUSSION

This study highlights the favorable hemodynamic profile of the OPCAB technique using simple suspension sutures as evidenced by reduced CO and low impact of the altered hemodynamic status on the renal tissue, which is evidenced by stable NGAL levels during surgery. We consistently observed lower CO during non-anatomical cardiac positions intraoperatively in patients who underwent off-pump CABG. The procedure was also not accompanied by increases in serum NGAL, creatinine, and urea concentrations, which are indicative of renal injury. Although there are some suggestions in the literature of a slight increase in renal enzymes during cardiac surgery in patients with OPCAB, our results appear to be contrary to the available recent studies^[7,9-11]^.

The CABG technique in a beating heart has been shown to be superior to the conventional methods in selected elderly patients^[10-12]^. Providing “ideal” vascular anastomosis is critical to the success of OPCAB. This is only possible with adequate stabilization and a bloodless anastomosis site. During OPCAB surgery, hemodynamic changes are inevitable due to the placement of the heart in different positions, the effects of the devices used for stabilization, and the temporary interruption of coronary blood flow. These hemodynamic changes have been reported in numerous studies^[13-14]^. Gründeman et al.^[15,16]^ showed that CO decreased more when the heart apex was moved out of the cavity and positioned vertically during OPCAB surgery in animal models. These hemodynamic effects were considered mainly to result from the disruption of the filling of the right ventricle. Although no mechanical stabilizers were used during surgery in the present study, the CO values obtained during all non-anatomical cardiac positions were significantly lower than the CO values obtained with the heart in the normal position. Ganesh Kumar et al. reported that despite the use of a deep pericardial suspension suture, mechanical stabilizer, and intracoronary shunt in patients undergoing OPCAB surgery, hemodynamic parameters obtained using the FloTrac system during non-anatomical cardiac positions were altered, and a significant decrease in CO levels compared to the normal cardiac position was observed^[2]^. This finding supports our view that the additional use of a mechanical stabilizer and intracoronary shunt during OPCAB surgery does not yield superior results in the hemodynamic parameters compared to the procedure performed using only a pericardial sling. One possible reason why the mechanical stabilizer does not provide additional benefits is that the decrease in CO is mainly due to an increase in the end-diastolic filling pressure of the right ventricle, as suggested by Gründeman et al.^[16]^ At this juncture, the important concern therefore is the effect of decreased CO during OPCAB surgery on renal functions.

In all reason, acute renal dysfunction is often attributed to hypoperfusion of the kidney due to impairment of CO during placement of the heart in different positions^[17-19]^. However, a drop in systemic blood pressure, venous congestion, and intra-abdominal pressure may be the hemodynamic parameters that are more strongly associated with worsening renal function in cardiac surgery patients^[20]^.

Conventional renal function tests such as serum creatinine and creatinine clearance are not considered to be sensitive indicators of early renal damage due to the size of renal reserve. Several biomarkers have been investigated with the aim of early detection of renal damage, among which NGAL has maintained its status as the most popular. Mishra et al.^[17]^ concluded that serum NGAL levels were an appropriate marker that can be used with high sensitivity and specificity in determining early acute renal failure following cardiac surgery^[17,18]^. Similarly, Fadel et al.^[19]^ reported that NGAL levels may be useful in the detection of early stage renal damage following CABG. Based on these findings, we decided to monitor NGAL levels instead of serum creatinine to identify changes in the renal blood supply. NGAL levels were determined at five different time periods in order to better detect any renal perfusion defect; of these, the level at the postoperative 4^th^ hour (T4) was relatively higher, but the difference was not statistically significant when compared to the NGAL values determined at other time points. In addition, there was no correlation between CO and NGAL values measured at the same corresponding time points. Urea and creatinine levels did not increase to pathological levels, indicating that acute renal failure did not develop in these patients.

OPCAB surgery performed using only a pericardial sling resulted in reduced CO values at non-anatomical positions of the heart. However, this did not result in incomplete revascularization in any patient, nor did it necessitate a transition to CPB. Moreover, none of the patients received a coronary shunt in the current study. Thus, in OPCAB surgeries, procedures such as more extensive opening of the right pleura to facilitate right ventricular filling and placing the patient in the Trendelenburg position may be beneficial in ensuring adequate CO. Although there was a significant decrease in CO when only the pericardial sling method was employed, the procedure was not accompanied by renal ischemia.

### Limitations

The low number of participants, the lack of a clear relationship between serum NGAL levels and other clinical parameters (*e.g*. age), and most importantly, the lack of concrete clinical data pertaining to NGAL are the primary limiting factors of our study. In addition, while the presence of adequate/sufficient renal perfusion was evaluated in the context of serum NGAL levels, lack of evaluation of the effect of the current method on cerebral blood flow constitutes another limitation. Further studies will indicate whether kidney damage can be detected early and whether OPCAB can be performed using even simpler methods.

## CONCLUSION

The OPCAB technique carried out without the use of a mechanical stabilizer was associated with safe CO levels, full revascularization, and no incidence of renal ischemia. The absence of a significant increase in serum NGAL values in relation to the number and/or variety of coronary anastomoses or the duration of surgery and the absence of acute renal failure in any patient in the days following surgery are indicative of the safety of this surgical technique performed without the use of a mechanical stabilizer or coronary shunt. In addition, with adequate surgical experience, as well as timely and appropriate technical manipulations, the success rate of the method may be increased.

**Table:** 

Authors' roles & responsibilities	
SS	Substantial contributions to the conception or design of the work; or the acquisition, analysis, or interpretation of data for the work; drafting the work or revising it critically for important intellectual content; agreement to be accountable for all aspects of the work in ensuring that questions related to the accuracy or integrity of any part of the work are appropriately investigated and resolved; final approval of the version to be published
ZT	Substantial contributions to the conception or design of the work; or the acquisition, analysis, or interpretation of data for the work; drafting the work or revising it critically for important intellectual content; agreement to be accountable for all aspects of the work in ensuring that questions related to the accuracy or integrity of any part of the work are appropriately investigated and resolved; final approval of the version to be published
KC	Substantial contributions to the conception or design of the work; or the acquisition, analysis, or interpretation of data for the work; drafting the work or revising it critically for important intellectual content; agreement to be accountable for all aspects of the work in ensuring that questions related to the accuracy or integrity of any part of the work are appropriately investigated and resolved; final approval of the version to be published
KO	Substantial contributions to the conception or design of the work; or the acquisition, analysis, or interpretation of data for the work; drafting the work or revising it critically for important intellectual content; agreement to be accountable for all aspects of the work in ensuring that questions related to the accuracy or integrity of any part of the work are appropriately investigated and resolved; final approval of the version to be published
UK	Substantial contributions to the conception or design of the work; or the acquisition, analysis, or interpretation of data for the work; drafting the work or revising it critically for important intellectual content; agreement to be accountable for all aspects of the work in ensuring that questions related to the accuracy or integrity of any part of the work are appropriately investigated and resolved; final approval of the version to be published
